# Low-molecular-weight heparin-induced thrombocytopenia with multisite embolism: successful management with argatroban and dabigatran – a case report and literature review

**DOI:** 10.3389/fphar.2025.1573840

**Published:** 2025-07-11

**Authors:** Huixin Zhao, Xiaowan Tang, Xitao Song

**Affiliations:** ^1^ Department of Pharmacy, North China University of Science and Technology Afffliated Hospital, Tangshan, China; ^2^ Department of Pharmacy, Peking Union Medical College Hospital, Chinese Academy of Medical Science and Peking Union Medical College, Beijing, China; ^3^ Department of Vascular Surgery, Peking Union Medical College Hospital, Chinese Academy of Medical Science and Peking Union Medical College, Beijing, China

**Keywords:** low-molecular-weight heparin (LMWH), heparin-induced thrombocytopenia (HIT), argatroban, dabigatran etexilate, anticoagulation management

## Abstract

Heparin-induced thrombocytopenia (HIT) is a rare but potentially life-threatening complication, with an incidence ranging from approximately 0.2%–5.0%. The risk of HIT associated with low-molecular-weight heparin (LMWH) is nearly ten times lower than that of unfractionated heparin (UFH). However, LMWH can still induce severe thrombocytopenia and thromboembolic events. This study presents a rare case of LMWH-induced severe type II thrombocytopenia complicated by multiple thromboembolic events. An elderly patient developed HIT following LMWH administration and experienced worsening embolic symptoms after platelet transfusion. The patient received timely discontinuation of heparin analogues and initiation of argatroban therapy with close monitoring of activated partial thromboplastin time (APTT). This was followed by a transition to dabigatran etexilate, which successfully prevented life-threatening embolic complications, limb amputation, and mortality. This case underscores the importance of maintaining a high level of clinical vigilance despite the rarity of LMWH-induced HIT. Once HIT is diagnosed, all forms of heparin should be discontinued immediately, and the decision to administer platelet transfusion should be made with caution to prevent exacerbation of thrombosis. This study provides valuable insights into the early recognition and optimal management of LMWH-induced HIT.

## Introduction

HIT is an adverse drug reaction mediated by platelet-activating antibodies, characterized by a significant decrease in platelet count during clinical treatment and an exceptionally high risk of thrombosis (Marcucci et al., 2021). There are two main types of HIT: Type I HIT is non-immune-mediated thrombocytopenia, usually caused by direct platelet activation by heparin, and is typically mild and self-limiting. Type II HIT, on the other hand, is an immune-mediated thrombocytopenia resulting from the formation of immune complexes between heparin and platelet factor 4 (PF4), which activate platelets and trigger thrombosis. In severe cases, this can lead to venous and arterial thrombotic events with a high mortality rate (Mongirdienė et al., 2023). In contemporary medical terminology, as well as in this study, the term “HIT” refers exclusively to type II HIT (Xu, 2018). The incidence of HIT with thrombosis (HITT) ranges from 20% to 64%, with severe cases leading to deep vein thrombosis, pulmonary embolism (PE), and even ischemic gangrene of the limbs. The associated risk of amputation and mortality can be as high as 20%–30% (Mongirdienė et al., 2023).

LMWH is derived from the depolymerization of UFH and is widely used in the prophylaxis and treatment of deep vein thrombosis (Hirsh et al., 2001). Compared to UFH, LMWH carries a significantly lower risk of HIT. The overall incidence of HIT in patients receiving UFH is estimated to be 0.2%–5.0%, whereas the incidence of LMWH-associated HIT is approximately one-tenth of that observed with UFH (Elshoury et al., 2015). However, despite its lower risk, LMWH-induced HIT can still lead to severe thrombotic events, particularly if not promptly recognized and appropriately managed. Moreover, platelet transfusions in such cases may further exacerbate thrombosis and increase the risk of mortality.

In recent years, with the widespread use of anticoagulant drugs and advancements in HIT diagnostic techniques, early identification and precise management of HIT have become a key focus of research (Maličev, 2020). The pathophysiological mechanism of HIT involves a complex immune response, and its diagnosis requires a combination of clinical assessment and laboratory testing, such as the 4T’s scoring system and specific antibody assays (Joseph et al., 2019; Nagler et al., 2016). Currently, non-heparin anticoagulants (e.g., argatroban, fondaparinux) have become the primary treatment options for HIT. However, a comprehensive evaluation of the optimal dosage of different anticoagulants, timing of treatment initiation, and long-term prognostic management remains lacking (Koster et al., 2022). Additionally, in patients with severe HIT, the applicability of platelet transfusion and the optimization of anticoagulation strategies to mitigate the risk of complications remain significant clinical challenges (Cuker et al., 2018).

In this paper, we present a rare case of LMWH-induced severe type II HIT with multisite thrombosis, analyze the diagnostic and therapeutic process, and discuss strategies for early identification, accurate diagnosis, and individualized treatment of HIT. The aim of this study is to summarize and optimize key aspects of existing HIT management, provide a reference for clinical decision-making in similar cases, and examine the impact of inappropriate therapeutic choices on patient prognosis. Furthermore, we offer targeted recommendations for the prevention and treatment of HIT-related complications.

### Case description

A 70-year-old female patient (height: 160 cm, weight: 65 kg) received conservative treatment for a bone fracture in September 2024. In October, she developed bilateral lower limb edema of unknown cause, accompanied by pain but without other significant discomfort. She sought medical attention at an external hospital, where ultrasound examination suggested deep vein thrombosis (DVT) involving the right common iliac vein, internal iliac vein, external iliac vein, and inferior vena cava. On October 18, she underwent inferior vena cava (IVC) filter implantation and was initiated on thrombolytic therapy and LMWH anticoagulation. However, on October 27, her platelet count dropped sharply to 20 × 10^9^/L, prompting the discontinuation of heparin and isolated platelet collection. By October 28, her platelet count had further declined to 11 × 10^9^/L, and she exhibited progressive hemoglobin reduction along with transient loss of consciousness. On October 29, her anticoagulation regimen was switched to rivaroxaban 10 mg once daily (QD). Subsequently, she developed coldness and pain in the right foot and was admitted to the emergency department of our hospital on November 2 due to an unclear etiology of the sudden platelet decline and a critical condition. After admission, vascular ultrasound revealed extensive atherosclerosis with plaque formation in the lower limb arteries, with a possible occlusion of the right superficial femoral artery and reduced flow velocity in the right dorsalis pedis artery. Atherosclerosis and plaque formation were also observed in the bilateral iliac arteries. Deep vein ultrasound suggested thrombosis in the bilateral common femoral veins, superficial femoral veins, calf intermuscular veins, and the left popliteal vein. A hypoechoic area in the right calf intermuscular region required further evaluation to exclude hematoma. Iliac vein ultrasound indicated possible thrombus formation in the bilateral external iliac veins. Inferior vena cava ultrasound showed hyperechoic regions suggestive of thrombosis. Physical examination revealed a body temperature of 36.6°C, pulse rate of 81 beats per minute, blood pressure of 140/67 mmHg, respiratory rate of 18 breaths per minute, and SpO_2_ of 95%. Emergency laboratory tests showed WBC 7.41 × 10^9^/L, NEUT% 74.1%, HGB 72 g/L, PLT 28 × 10^9^/L, CRP/hs-CRP 165.50 mg/L, NT-proBNP 522 pg/mL, PT 15.3 s, APTT 33.7 s, and D-dimer 16.23 mg/L FEU. The patient had a history of hypertension and diabetes mellitus but no known drug allergies. The admission diagnosis was as follows: (1) thrombocytopenia; (2) multiple venous thromboses involving the inferior vena cava, bilateral external iliac veins, bilateral common femoral veins, superficial femoral veins, intermuscular veins of the calf, and the left popliteal vein; (3) status post inferior vena cava filter implantation; (4) intra-aortic thrombosis, suspected intermuscular hematoma in the right calf, and atherosclerosis with plaque formation affecting the bilateral common femoral, anterior tibial, left superficial femoral, popliteal, and right posterior tibial arteries, with possible occlusion of the right superficial femoral artery; (5) hypertension; and (6) diabetes mellitus.

**FIGURE 1:**
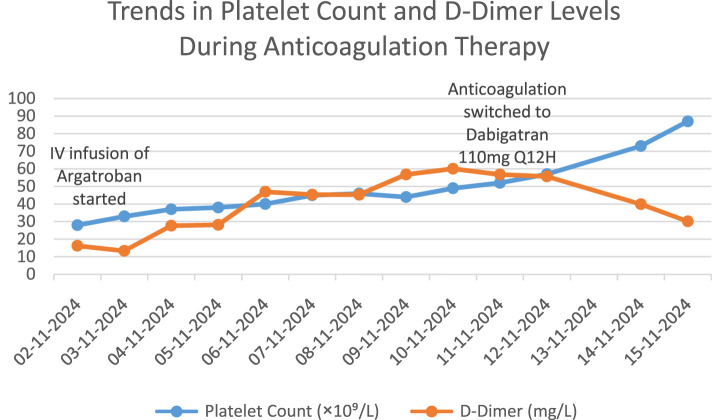
Trends in platelet count and D-Dimer levels during anticoagulation therapy.

After admission, comprehensive examinations revealed acute unexplained thrombocytopenia accompanied by multisite thrombosis, indicating a critical and complex condition. Following a multidisciplinary consultation with the clinical pharmacist and the vascular surgery department, the clinical pharmacist determined a temporal association between the onset of thrombocytopenia and the use of low-molecular-weight heparin (LMWH). Given the patient’s 4Tʼs score of 7, heparin-induced thrombocytopenia (HIT) was strongly suspected. Based on this assessment, the use of heparin analogues was avoided, and anticoagulation with argatroban (Haritet, 20 mL: 10 mg) was recommended. The prescribed regimen was 20 mL/day diluted in 40 mL of saline and administered intravenously at a rate of 0.7 mL/h. During this period, the patient was closely monitored for potential hemorrhagic risks and thromboembolic complications, including cerebral infarction, ischemia of the limbs, gastrointestinal tract, liver, spleen, and kidneys (Xu, 2018). The vascular surgery department concluded that the patient’s thrombocytopenia was a contraindication for surgery and concurred with the treatment plan proposed by the clinical pharmacist. It was recommended to maintain activated partial thromboplastin time (APTT) within the range of 40–50 s (Pollak, 2019) and to perform an HIT antibody test for diagnostic confirmation. The test results on November 4 indicated a platelet count (PLT) of 37 × 10^9^/L and a positive HIT antibody (HIT-Ab) result of 6.2 U/mL (Xu, 2021). Based on these findings, a diagnosis of HIT was confirmed. Although a dynamic review showed a gradual increase in platelet count, the absolute value remained low. Therefore, continued anticoagulation with argatroban was recommended, with APTT maintained in the target range and close monitoring of platelet count and coagulation function. Vigilance for signs of active bleeding and hemorrhagic shock was emphasized. It was advised that once platelet levels recovered to >50 × 10^9^/L, a transition to dabigatran for ongoing anticoagulation could be considered (Kearon et al., 2016). On November 6, argatroban infusion was continued at 0.8 mL/h, maintaining APTT within the target range of 40–50 s. Over the following days, the patientʼs platelet count increased steadily, By November 11, PLT had risen to 52 × 10^9^/L, with a corresponding D-dimer level of 56.79 mg/L FEU. On November 12, HGB was 80 g/L, PLT increased to 57 × 10^9^/L, and D-dimer was 55.69 mg/L FEU. By November 14, HGB had risen to 96 g/L, PLT to 73 × 10^9^/L, and D-dimer had decreased to 30.60 mg/L FEU. On November 15, PLT reached 87 × 10^9^/L, demonstrating a clear trend of platelet recovery. On November 11, after at least 5 days of intravenous anticoagulation, dabigatran etexilate (110 mg q12 h) was initiated (Kearon et al., 2016). The clinical pharmacist advised continuous monitoring of D-dimer levels following the switch to dabigatran. If D-dimer showed an upward trend suggestive of inadequate anticoagulation, dose adjustment and bleeding surveillance would be necessary. The chronological changes in PLT and D-dimer levels after admission are illustrated in Figure 1. Upon stabilization, the patient was discharged with instructions to avoid all heparin analogues. Given the ongoing platelet recovery, stable hemoglobin levels, and no significant abnormalities in other laboratory parameters, she was transferred to a local hospital for further management.

Post-discharge follow-up indicated that the patient remained stable while continuing dabigatran etexilate therapy. During a follow-up visit on 21 January 2025, coagulation indices were found to be within normal limits, except for an elevated thrombin time (TT) of 120 s. Concerned about the elevated TT, the vascular surgeon consulted a clinical pharmacist regarding the necessity of discontinuing dabigatran etexilate. After evaluation, the clinical pharmacist determined that the increased TT was a known pharmacological effect of dabigatran and, in isolation, did not imply an increased bleeding risk. Therefore, it was recommended to continue dabigatran therapy while implementing enhanced follow-up monitoring to ensure both the safety and efficacy of anticoagulation treatment.

## Discussion

### Pathogenesis and diagnosis of HIT

HIT is a severe complication associated with heparin therapy (Li et al., 2023). The underlying mechanism of thrombocytopenia and thrombosis in HIT primarily involves the formation of the PF4-heparin-IgG complex, which binds to the FcγRIIa receptor on the platelet surface. This interaction leads to receptor cross-linking, resulting in excessive platelet activation, the release of platelet granule contents, and the generation of procoagulant particles that promote thrombin formation. Consequently, endothelial cells, neutrophils, and monocytes become activated, further exacerbating the prothrombotic state (Joseph et al., 2019). Although HIT-associated antibody formation is relatively common, only 0.2%–3.0% of patients progress to clinically significant HIT. The condition is primarily characterized by thrombocytopenia and/or thrombosis, which can manifest as arterial, venous, or microvascular thrombosis. Thrombosis is the most severe complication of HIT, significantly increasing morbidity and mortality, with approximately 50% of patients with acute HIT developing new thrombotic events (Nie et al., 2024). A study by Goel et al. (2015) demonstrated that the risk of thrombosis in patients with HIT is closely related to the degree of thrombocytopenia and that platelet transfusion is significantly associated with an increased risk of arterial thrombosis. The clinical course of our patient, who developed extensive arterial thrombosis shortly after platelet transfusion, aligns with these findings and further underscores the strong association between platelet activation and thrombosis in HIT.

The diagnosis of HIT is based on a combination of clinical scoring systems and laboratory tests. Current guidelines recommend that all patients suspected of HIT undergo 4T’s scoring before laboratory testing. Further testing with immunoassays or functional assays is generally not recommended for individuals with a low 4T’s score. However, for patients with an intermediate to high 4T’s score, confirmatory testing with immunoassays is required to establish the diagnosis (Joseph et al., 2019; Nagler et al., 2016).

Our patient’s platelet count (PLT) dropped to as low as 11 × 10^9^/L, which is classified as severe thrombocytopenia according to the Chinese expert consensus (Chinese Expert Consensus Group on Emergency Management of Adult Thrombocytopenia et al., 2022). During the pharmacovigilance assessment, the clinical pharmacist determined that the most likely cause of the patient’s platelet decline was LMWH sodium. The patient exhibited a significant decrease in platelet count on day 9 following the initiation of LMWH therapy, which falls within the typical onset window for HIT (5–10 days) (Gruel et al., 2020). Based on the 4T’s scoring system, the patient presented with severe thrombocytopenia, and the timing of platelet decline closely corresponded with LMWH administration, as evidenced by a marked drop on day 9. Additionally, the patient developed multi-site thrombosis without any other apparent cause of thrombocytopenia. With a 4T’s score of 7, the likelihood of drug-induced HIT was strongly suspected. This diagnosis was subsequently confirmed by a positive HIT antibody test, establishing a definitive diagnosis of severe type II HIT induced by LMWH sodium.

In addition to the 4T’s score and HIT antibody testing, functional assays such as the serotonin release assay (SRA) remain the gold standard for the definitive diagnosis of HIT. The SRA has a sensitivity and specificity of approximately 95% and confirms the pathogenicity of HIT antibodies by evaluating platelet activation in response to heparin (May and Cuker, 2024). Due to its technical complexity, reliance on specialized laboratory conditions, and delayed turnaround time, the SRA is typically used as a supplementary test in cases where immunoassay results are inconclusive or when clinical suspicion remains high. Notably, some studies have reported that anti-PF4 antibody detection may be influenced by factors such as altered antigenic complexes following SARS-CoV-2 infection (COVID-19) or vaccination, potentially leading to false-negative or atypical results in individual cases (Uzun et al., 2022). Therefore, in patients with high clinical suspicion of HIT, a comprehensive assessment combining functional testing and clinical scoring remains essential.

### Treatment of HIT

Once HIT is suspected or diagnosed, all heparin analogues must be immediately discontinued and replaced with non-heparin anticoagulants (Cuker et al., 2018). Initial anticoagulant therapy for HIT typically involves parenterally administered direct thrombin inhibitors (e.g., bivalirudin, argatroban) or indirect factor Xa inhibitors, such as fondaparinux, which should be administered at therapeutic doses rather than prophylactic doses (Joseph et al., 2019). For critically ill patients, those at high risk of bleeding, or those who may require urgent surgery, argatroban or bivalirudin is generally preferred due to their shorter half-life, which allows for rapid adjustment of anticoagulation intensity, thereby minimizing the risk of bleeding (Cuker et al., 2018). Transitioning to oral anticoagulants for long-term management may be considered once platelet counts return to a safe level. In recent years, the use of direct oral anticoagulants (DOACs) in HIT treatment has gained increasing attention. Studies have demonstrated that rivaroxaban can be effective for both initial and maintenance therapy in HIT (Linkins et al., 2016; Chen et al., 2021). The American Society of Hematology (ASH) guidelines also recommend the use of DOACs in HIT treatment (Cuker et al., 2018).

However, in this case, a prophylactic dose of rivaroxaban (10 mg once daily) was administered during the initial treatment phase of HIT, which is not appropriate for therapeutic anticoagulation. The 10 mg dose is typically indicated for venous thromboembolism (VTE) prophylaxis following orthopedic surgery or for secondary prevention in patients at high risk of recurrent DVT or PE. In contrast, therapeutic management of HIT generally requires an initial dose of rivaroxaban 15 mg twice daily during the acute phase, followed by 20 mg once daily after 3 weeks, with anticoagulation maintained for at least 3 months to reduce the risk of recurrent thrombosis (Skelley et al., 2016). Additionally, some studies have reported favorable outcomes in HIT patients treated with rivaroxaban 20 mg twice daily, highlighting the importance of individualized dosing strategies (Abouchakra et al., 2015).

For patients with severe HIT with multiple thrombotic sites, the initial treatment regimen should prioritize intravenous anticoagulation, followed by a transition to oral anticoagulant therapy once the patient stabilizes (Cuker et al., 2018). In this case, following the confirmed diagnosis of HIT, LMWH was immediately discontinued, and the patient was started on intravenous argatroban infusion, with dose adjustments made to maintain APTT within the target range of 40–50 s. This approach effectively reduced the risk of HIT-related thrombosis (Fisser et al., 2021; Koster et al., 2022). The gradual rebound in platelet count during treatment further indicated the efficacy of the anticoagulation strategy. Argatroban is a short-acting direct thrombin inhibitor that exerts its anticoagulant effect by directly inhibiting thrombin, thereby suppressing fibrin formation, platelet aggregation, and the activity of coagulation factors V, VIII, XIII, and protein C. It has the pharmacokinetic advantage of a short half-life, allowing for precise dose titration based on APTT monitoring and rapid discontinuation in the event of bleeding complications (Han and Wang, 2021). According to the 2018 ASH guidelines (Cuker et al., 2018), the preferred treatment for acute HIT includes non-heparin anticoagulants such as argatroban, bivalirudin, danaparoid, fondaparinux, and DOACs. Argatroban or bivalirudin may be particularly preferred in critically ill patients, those with an increased risk of bleeding, or those who may require urgent surgery due to their short duration of action and ease of control. In a retrospective Bayesian network meta-analysis (Colarossi et al., 2021), argatroban demonstrated superior overall efficacy compared with other commonly used anticoagulants in HIT, including lepirudin, danaparoid, and bivalirudin, especially in selected patient populations.

During the transition from intravenous to oral anticoagulation in patients with HIT, medication selection should take into account mechanistic continuity, pharmacokinetic characteristics, and patient-specific factors. According to the ASH guidelines (Cuker et al., 2018), DOACs, such as dabigatran, rivaroxaban, and apixaban, are preferred over vitamin K antagonists (VKAs). The choice of a specific DOAC should be guided by drug properties, clinical experience, and individualized patient needs. In this case, dabigatran etexilate was selected over other DOACs primarily due to its mechanism of action, which aligns with that of the intravenous agent argatroban-a direct thrombin inhibitor-facilitating a seamless pharmacological transition (Warkentin et al., 2017). Furthermore, the ASH guidelines support the use of dabigatran after at least 5 days of parenteral non-heparin anticoagulant therapy, provided the platelet count has recovered to a safe level. Dabigatran may therefore offer particular advantages for patients transitioning from intravenous thrombin inhibitors to oral anticoagulants. Notably, this patient had previously received rivaroxaban 10 mg once daily at an outside facility, a dose that did not meet the recommended therapeutic intensity for HIT and failed to control thrombotic progression. Based on the need for mechanistic continuity and in consideration of prior treatment failure, oral dabigatran etexilate was selected, with favorable clinical outcomes. This treatment approach is consistent with findings by Nasiripour et al. (Nasiripour et al., 2019), who reported that dabigatran can serve as a safe and effective anticoagulant alternative in HIT, supporting both thrombosis prevention and platelet recovery.

In addition, dabigatran etexilate has a significant impact on TT, typically resulting in marked prolongation. However, an isolated elevation in TT is not necessarily indicative of an increased bleeding risk; rather, it may serve as an indirect marker of dabigatran’s anticoagulant activity (Liu et al., 2022). TT measures the time required for fibrin formation after the addition of thrombin to plasma. As a direct thrombin inhibitor, dabigatran binds directly to thrombin (factor IIa), thereby inhibiting its activity and prolonging the TT. TT is highly sensitive even at low plasma concentrations of dabigatran, and its prolongation does not correlate linearly with bleeding risk. A study by Shaw et al. (Shaw et al., 2022) demonstrated that TT values are positively correlated with dabigatran plasma concentrations but should not be used as a standalone indicator for bleeding risk assessment. Therefore, current literature does not recommend using TT alone to guide dose adjustment or discontinuation; rather, it should be interpreted in the context of the patient’s clinical condition and other coagulation parameters. In this case, although the patient’s TT was elevated to 120 s, there were no signs of bleeding, and hemoglobin, APTT, and platelet counts remained stable—supporting the continuation of dabigatran therapy.

Current guidelines recommend at least 1 month of anticoagulation for isolated HIT, whereas HITT requires a minimum of 3 months of anticoagulation to reduce the risk of thrombotic recurrence and associated complications (Xu, 2018; Joseph et al., 2019). Based on this evidence, the clinical pharmacist recommended continuing dabigatran etexilate therapy for a full 3-month course, with enhanced follow-up monitoring, but without the need for dose adjustment. Through pharmacological monitoring by the clinical pharmacist, combined with an individualized anticoagulation strategy, the patient in this case achieved successful recovery and was discharged in stable condition. This case provides valuable clinical insights for the optimized management of HIT, emphasizing the importance of precision-based anticoagulation therapy.

### HIT and the appropriateness of platelet transfusion

Thrombocytopenia in HIT is primarily caused by platelet consumption and immune-mediated destruction, rather than by bone marrow suppression. According to the Chinese expert consensus (Xu, 2018), platelet transfusions should be administered with extreme caution in patients with HIT to avoid precipitating or exacerbating thrombotic events. Transfused platelets may enhance the immune-thrombotic cascade by increasing the number of activated platelets and providing additional binding sites for HIT antibodies, thereby elevating the risk of new thrombotic events (Goel et al., 2015). If transfusion is deemed necessary, it must be performed only after discontinuation of all heparin products and in conjunction with non-pharmacological haemostatic measures. The French Working Group on Perioperative Haemostasis (GIHP) (Gruel et al., 2020), in its guidelines on the diagnosis and management of HIT, also advises against routine platelet transfusion, citing lack of efficacy and the potential to promote thrombosis. Similarly, the ASH guidelines do not recommend platelet transfusion except in cases of life-threatening bleeding or the need for urgent surgical intervention (Cuker et al., 2018). This view is supported by a meta-analysis conducted by Morgan et al. (Morgan et al., 2020), which demonstrated a significant association between platelet transfusion and increased risk of new thrombotic events in HIT patients. In the present case, the patient’s condition deteriorated rapidly following platelet transfusion at an outside facility, subsequently developing multi-site thrombosis—further supporting the potential harms of platelet transfusion in the context of HIT. Therefore, platelet transfusion in HIT should be strictly reserved for well-defined clinical indications to minimize the risk of thromboembolic complications.

## Refined summary

This study presents a rare case of low molecular weight heparin-induced heparin-induced thrombocytopenia (HIT) with multisite thrombosis. Through early recognition, laboratory confirmation, prompt discontinuation of heparin analogues, anticoagulation with argatroban, and transition to dabigatran etexilate, fatal embolic complications were successfully avoided. Based on this case, we derive the following key clinical insights:

Early recognition of HIT is critical–When unexplained thrombocytopenia occurs during anticoagulation therapy, particularly when accompanied by thrombosis, HIT should be considered as a potential diagnosis. Timely laboratory testing is essential for confirmation.

Argatroban is the preferred anticoagulant in the acute phase of HIT–Close monitoring of APTT is necessary to balance anticoagulant efficacy with bleeding risk.

Dabigatran is a viable long-term anticoagulation option for HIT patients–While further studies are needed to establish its role in HIT management, this case demonstrates that dabigatran etexilate can be a safe and effective alternative.

Platelet transfusion should be approached with caution–Routine platelet transfusion is not recommended to prevent exacerbation of thrombosis, unless there is life-threatening bleeding that necessitates intervention.

This case provides valuable clinical insights into the diagnosis and management of HIT, offering a reference for optimizing treatment strategies. Moving forward, larger-scale studies are needed to further explore optimal anticoagulation approaches in HIT and evaluate the efficacy of novel anticoagulants in improving patient outcomes and prognosis.

## Data Availability

The original contributions presented in the study are included in the article/supplementary material, further inquiries can be directed to the corresponding author.
